# P24 Auditing antibiotic course lengths for the management of community-acquired pneumonia and hospital-acquired pneumonia against current NICE guidance

**DOI:** 10.1093/jacamr/dlad066.028

**Published:** 2023-06-14

**Authors:** Elaine Chua, Daniel Hearsey

**Affiliations:** Pharmacy Department, Royal Cornwall Hospitals NHS Trust, Truro, UK; Pharmacy Department, Royal Cornwall Hospitals NHS Trust, Truro, UK

## Abstract

**Background:**

Protracted antibiotic course lengths for the treatment of community-acquired pneumonia (CAP) and hospital-acquired pneumonia (HAP) contribute to increased risk of antibiotic resistance.^1^ Optimizing antibiotic course length in line with the current national guidance improves patient safety and overall cost savings.^1^ NICE guidelines state that antibiotic treatment should be stopped at day 5 if patients were afebrile for <48 hours with one or no sign of clinical instability, defined as systolic blood pressure < 90 mmHg, heart rate > 100/minute, respiratory rate > 24/minute, arterial oxygen saturation < 90% or oxygen partial pressure > 60 mmHg in room air.^2^

**Objectives:**

To assess the current prescribing practice for antibiotic course lengths of CAP and HAP across medical specialties at RCHT.

**Methods:**

A list of patients discharged from medical specialities (respiratory, cardiology, nephrology, gastroenterology, endocrinology and geriatrics) between 1 August and 1 September 2022 with the coded indication of CAP or HAP was requested from the RCHT Informatics Team. Inclusion criteria were any hospitalized patient aged ≥18 years, with a CAP/HAP diagnosis with pulmonary infiltrate on chest X-ray plus at least one symptom including cough, fever, dyspnoea and chest pain. Patients were excluded if having chronic immunosuppression; HIV positive; living in a nursing/care home, onsite subacute care unit or intensive therapy unit (ITU) or palliative care unit within last 14 days; having taken antibiotics 30 days before admission; requiring a longer duration of therapy because of an uncommon cause; requiring a chest tube or having had a condition complicated by extrapulmonary infection.^1^ For each patient, the patient’s vital signs at day 5 of antibiotic course and the total antibiotic course lengths were recorded.

**Results:**

A total of 27 patients were assessed for eligibility; of these, 22 patients (81%) met the inclusion criteria, and 5 patients were excluded. Among those excluded, two recently stepped down from ITU, two lived in a nursing/care home and one had taken antibiotics 30 days before admission. Of the 22 patients, 10 patients (45%) received 5 or 6 days of antibiotics for CAP/HAP. The remaining 12 patients (55%) received courses between 7 to 10 days despite meeting the criteria for stopping antibiotics at 5 days. The mean and median of antibiotic course length including all specialities were 6.5 and 6.3 days, respectively.

**Conclusions:**

This audit demonstrates that almost half of patients with CAP /HAP are being managed with antibiotic courses of fewer than 7 days. Further prescriber and pharmacy team education sessions on appropriate course lengths are needed. There is consideration for an alert notification on EPMA which reminds prescribers to check patient’s vital signs on day 5 when prescribed an antibiotic for the treatment of CAP and HAP. Recommendation would be to reaudit with a longer data collection timeframe and larger sample size.
